# Deepfake attack prevention using steganography GANs

**DOI:** 10.7717/peerj-cs.1125

**Published:** 2022-10-20

**Authors:** Iram Noreen, Muhammad Shahid Muneer, Saira Gillani

**Affiliations:** Department of Computer Science, Bahria University, Islamabad, Lahore Campus, Pakistan

**Keywords:** Deepfake, Encryption, Watermark, Deep learning, Prevention, CNN, Steganographic, GANs

## Abstract

**Background:**

Deepfakes are fake images or videos generated by deep learning algorithms. Ongoing progress in deep learning techniques like auto-encoders and generative adversarial networks (GANs) is approaching a level that makes deepfake detection ideally impossible. A deepfake is created by swapping videos, images, or audio with the target, consequently raising digital media threats over the internet. Much work has been done to detect deepfake videos through feature detection using a convolutional neural network (CNN), recurrent neural network (RNN), and spatiotemporal CNN. However, these techniques are not effective in the future due to continuous improvements in GANs. Style GANs can create fake videos with high accuracy that cannot be easily detected. Hence, deepfake prevention is the need of the hour rather than just mere detection.

**Methods:**

Recently, blockchain-based ownership methods, image tags, and watermarks in video frames have been used to prevent deepfake. However, this process is not fully functional. An image frame could be faked by copying watermarks and reusing them to create a deepfake. In this research, an enhanced modified version of the steganography technique RivaGAN is used to address the issue. The proposed approach encodes watermarks into features of the video frames by training an “attention model” with the ReLU activation function to achieve a fast learning rate.

**Results:**

The proposed attention-generating approach has been validated with multiple activation functions and learning rates. It achieved 99.7% accuracy in embedding watermarks into the frames of the video. After generating the attention model, the generative adversarial network has trained using DeepFaceLab 2.0 and has tested the prevention of deepfake attacks using watermark embedded videos comprising 8,074 frames from different benchmark datasets. The proposed approach has acquired a 100% success rate in preventing deepfake attacks. Our code is available at https://github.com/shahidmuneer/deepfakes-watermarking-technique.

## Introduction

Data is the new oil of the world. Fake videos and images are increasing on the internet with the introduction of auto-encoders (Ballard, 1987) and generative adversarial networks (GANs) (Goodfellow, 2014). Deepfake is a method of swapping faces in images or videos with a target fake person. Manual detection of deepfakes by the human eye is difficult due to infusion in the target image. Consequently, it can pose a threat to celebrities, individuals, and political figures in their personal and professional lives because an attacker can fake the video for a malicious purpose (Hasan & Salah, 2019). This fake content has raised concerns about the integrity and authenticity of digital information, *e.g*., audio, video, and image content on the internet.

User-friendly deepfake creation applications are easily available, and a naïve user can also create deepfake easily. Deepfake detection techniques originated in response to concerns about fake news, misleading, counterfeit images and videos. To combat deepfake threats to society, deepfake detection techniques have been proposed by several researchers (Nguyen et al., 1909). Convolutional neural network (CNN) (Nguyen et al., 1909) based detection has been used to train many videos to detect minor feature changes in fake videos. Other methods detect deepfakes by either detecting blurred features in an image or eye blinking (Jung, Kim & Kim, 2020) issues in a video. Besides CNN, recurrent neural network (RNN) (Guera & Delp, 2019) has also been used for deepfake detection. Another prominent detection approach uses blockchain and watermarking. A blockchain-based technique (Hasan & Salah, 2019) authenticates the ownership of the content. A watermark-based technique (Wang et al., 2020; Alattar, Sharma & Scriven, 2020) embeds an image with a message as a tag value. This tag value is verified to confirm if the video is tempered or not. However, mere detection methods are not effective to combat intractable and challenging deepfake threats (Karras, Laine & Aila, 2018). Deepfake is becoming increasingly real as a result of GANs. The real cure is prevention, not detection. Therefore, the need of the hour is to eliminate the chances of deepfake creation (Wang et al., 2020).

The following are the research questions addressed in this study:

1. How can encoder and decoder networks be used to prevent deepfakes?
2. How will watermarks help prevent deepfakes?

This study has investigated the deepfake attack and deepfake detection approaches and has proposed an encryption-based deep learning approach for the prevention of deepfake attacks. The main contributions of this study are summarized as follows:

1. A GANs-based deep learning approach is proposed for deepfake prevention.
2. Watermark encryption is combined with deep learning steganography.
3. The proposed approach provides 100% deepfake prevention.

The remaining article is structured as follows: “Materials and Methods” discusses the related work and details of the proposed approach. “Results” discusses the results of implementation on bench-mark datasets. “Discussion” provides insight into performance evaluation and its comparison with state-of-the-art approaches. “Conclusion” concludes the discussion with the future work intended to further improve and extend this research.

## Materials and Methods

### Related work

Deepfake videos are generated by deep learning using a large amount of data in face-swapping. The more samples, the more realistic deepfake videos are generated. The Obama video was created by projecting the video for more than 56 h for a sample recording to make it realistic (Media Update, 2020; Floridi, 2018; Sultanov, Shevtsov & Nikolaev, 2020). Commonly, auto-encoders (Ballard, 1987) have been used to create deep fakes. In this method, the latent vector from image “*A*” is fed to the decoder of image “*B*”. The image generated from the decoder is swapped with a faked face image. FaceSwap (torzdf, 2014) uses auto-encoders to create deep fakes. Face-swap GAN (Shaoanlu, 2018) uses GANs to create precise deepfakes. The latent vector from the face “*A*” is generated and compared to generative models till the loss of both networks becomes equal. This newly generated fake image is then fed to the decoder network. DeepFaceLab (iperov, 2018) is an extended version of FaceSwap and generates high-quality, precise deepfakes. It includes either Multi-Task Cascaded (MTCNN) (Zhang et al., 2016), dlib (King, 2002), or manual methods for face extraction. The extracted face is then used to generate a deepfake using GANs or auto-encoder. The DFaker tool uses auto-encoders with the Keras loss function ‘Difference of Structural Similarity Index’ (DSSIM) (King, 2002). StyleGAN (torzdf, 2014; Yang & Qiao, 2021) is used to create realistic and high-quality deepfakes. Another creation tool, StarGAN (Choi et al., 2018), is used for image-to-image translation. It creates deepfakes of a single image with different emotions, *i.e*., happy, sad, or angry expressions.

Besides deepfake creation, several deepfake detection methods exist to identify or detect deepfakes. Image-based feature detectors (Wu et al., 2020) are used to detect deepfakes using histogram oriented gradients by measuring the feature discontinuities. Different deep learning models, *i.e*., CNN (Nguyen et al., 1909), RNN (Guera & Delp, 2019), *etc*., are used to detect deepfakes. Hongmeng et al. (2020) applied Gaussian blur to remove details that aren’t required to detect deepfakes. After compressing, videos are classified using the Resnet50 model. Spatiotemporal (de Lima et al., 2020) neural networks are also used to detect deepfakes by extracting only targeted features and classifying deepfakes using different classifiers, *i.e*., Discrete Fourier Transform (DFT), Recursive Cortical Network (RCN), 3D Deeper Residual Model (R3D), *etc*. Guarnera, Giudice & Battiato (2020) discovered Generative Adversarial Nets by applying a kernel just like a convolutional network but in reversed order. This process of image creation is different from the process of cameras. These traces can be examined and verified using the expectation-maximization algorithm (Moon, 1996). This gives over 90% of accuracy in detecting deepfakes. FaceNet (Wu et al., 2020) generates a vector representation of the face. It is subjected to Support Vector Regression (SVM), Gradient Boosting Decision Tree (GBDT), or Logistic to classify deepfakes. Ding et al. (2020) applied CNN to rank the deepfake detection using subjective assessment by web users.

The Scale Invariant Feature Transform (SIFT) algorithm (Dordevic, Milivojevic & Gavrovska, 2019) and eyebrow matching (Nguyen & Derakhshani, 2020) are also used in deepfake detection to calculate a match error. Nirkin et al. (2021) used the Dual Short Face Detector (DSFD) (Li et al., 2019) method to first capture the segmentation of the face in the video. Later, they used Xception Layers (Chollet, 2017) with inception architecture (Szegedy et al., 2017) and trained with the VGGface2 (Cao et al., 2018) dataset to detect deepfakes. Tariq, Lee & Woo (2021) identified that the detection models lack frame relationships during deepfake detection, hence missing frame change inconsistencies, *i.e*., changes in brightness, eye size, eyebrows, and lips. This phenomenon makes rendering unnatural. They used a convolutional LSTM residual network to capture the difference between real and fake frames for inter and intra-frame consistencies.

XceptionNet has been the best-performing network in the deepfake detection challenge (Deepfake Detection Challenge, 2020). Korshunov & Marcel (2022) modified XceptoinNet by replacing the final layer with a fully connected layer with a sigmoid activation function for the classification of deepfakes. Furthermore, they used EfficientNet Variant B4 for the detection of fake images or videos. Roy et al. (2022) first trained attention on the dataset to get the most prominent features of the video and then used I3D, 3D ResNet, and 3D ResNeXt to detect deepfakes. Kolagati, Priyadharshini & Mary Anita Rajam (2022) used the multi-layer perceptron to learn the difference between fake and real videos. Also, they used CNN to extract features. Furthermore, they combined models to detect deepfakes.

Hasan & Salah (2019) used blockchain-based applications to authenticate videos. The video is shared through a link on the Ethereum-based Interplanetary File System (IPFS). This video is then shared with the media through this link. Whenever this video is updated, or a pixel is updated, the server gets notified. Zhong & Shih (2020) embedded watermarks in images using deep learning. These watermarks aren’t visible but are extracted using extractor architecture (Zhong & Shih, 2020). The watermark is preserved, and whenever a deepfake is applied, watermarks change inner values. The variation in values decides if the video is fake or not. Alattar, Sharma & Scriven (2020) provided a web service to embed a watermark in video frames and video metadata is updated. When deepfake is performed, the watermark gets distorted. Upon testing the video with the provided web service, if the watermark is extractable from the video using the extractor algorithm, then the video is original; otherwise, deepfake is detected. Yu et al. (2020) created artificial fingerprints using generative adversarial networks (GANs) that are invisible to the naked eye. These fingerprints are embedded into the videos just like watermarks, as mentioned above. These fingerprints can be extracted using a decoder network. The extraction of the fingerprints determines the detection of deepfakes.

However, these detection methods may not always be effective due to ongoing advancements in GAN prevention methods that can be a potential solution for resolving deepfakes issues. Deepfake prevention is more concerned with preventing deepfake attacks rather than detecting them. In this method, the media is protected with an extra security layer. Either blockchain or watermarking can be used to prevent deepfakes. The need of the hour is to make fine use of new technologies and to post responsibly and ethically on digital media.

### Proposed approach

The proposed prevention approach comprises four stages. In the first phase pre-processed data is fed to 3D CNN to generate an “attention model”. In the second phase, a security method of encrypting watermarks is applied to video frames using a generative adversarial network and a convolutional neural network. This step protects information inside a video with a defined noise. An array of the watermark is then added to the features of an object by the GAN deep learning algorithm. An invisible watermark is embedded in video frames to prevent re-swapping of the same watermark. These watermarks are placed and transitioned through frames by GANs using the “attention model” created in the first phase.

Therefore, the once encrypted watermark is embedded in the video frames. It can be accessed and read-only if the “attention model” recipe is known. This “attention model” is the trained model with defined noise. Phase 3 shows that when an attacker tries to swap a fake image, the video will be validated with a watermarking extractor algorithm. The watermark extracting algorithm requires the same “attention model” to decode the watermark before the attacker can apply deepfake to the frame. Therefore, in phase 4, with the availability of the required “attention model”, the watermark presence will be verified using a presence probability score, *i.e*., the probability of watermark existence ensures that the video is not tempered, otherwise it is affected by deepfake. Thus, prevention occurs as the generated attention model by the proposed approach is not available to the attacker. In summary, the deepfake attacker cannot create a deepfake unless he has the “attention model” generated by the proposed approach. The absence of the “attention model” makes a deepfake attack impossible. The detailed procedure of the proposed approach is described in the following subsections.

### Dataset acquisition

Third-party public research datasets are used in this study. These datasets are available to developers and the scientific community for use in research and development. The UCF Action Recognition dataset (Soomro, Zamir & Shah, 2012) has been used for training watermark embedding. The UCF dataset comprises 101 actions with over 13K videos. Moreover, to embed watermarks and perform deepfakes A2D (Xu et al., 2015), the Hollywood2 dataset (Marszałek, Laptev & Schmid, 2009), and the TikTok trending videos dataset (van de Ven, 2020) are used. The main victim of deepfakes is social media websites because we believe what we see. Hence, we have used the trending TikTok video dataset to embed encrypted watermarks and then applied deepfakes to 5% of TikTok videos. TikTok videos are short-clip videos, so processing those videos will be the best way to ensure the prevention of deepfakes.

After embedding watermarks, the Hollywood2 dataset is used to apply deepfakes. An attempt to decode those watermarks will validate deepfake prevention. Table 1 shows the dataset summary. Table 2 shows the training, validation, and testing samples.

**Table 1:** Datasets summary.

Authors	Title	URL	Publishing year	Access date
Soomro, Zamir & Shah (2012)	UCF action data (Soomro, Zamir & Shah, 2012)	https://www.crcv.ucf.edu/data/UCF101.php	2012	2021
van de Ven (2020)	TikTok trending videos	https://www.kaggle.com/erikvdven/tiktok-trending-december-2020	2020	2021
Xu et al. (2015)	A2D actor dataset	https://web.eecs.umich.edu/~jjcorso/r/a2d/	2015	2021

**Table 2:** Details of training and validation and testing datasets.

Category	Dataset	Number of samples	Data format	Dimension
Training	UCF101 (Soomro, Zamir & Shah, 2012)	13,320	MP4, AVI	320 × 240
Validation	Hollywood (Marszałek, Laptev & Schmid, 2009)	196	MP4, AVI	320 × 240
Testing	TikTok (van de Ven, 2020)	1,000	Mp4	576 × 1,024

### Data pre-processing

**Figure 1:**
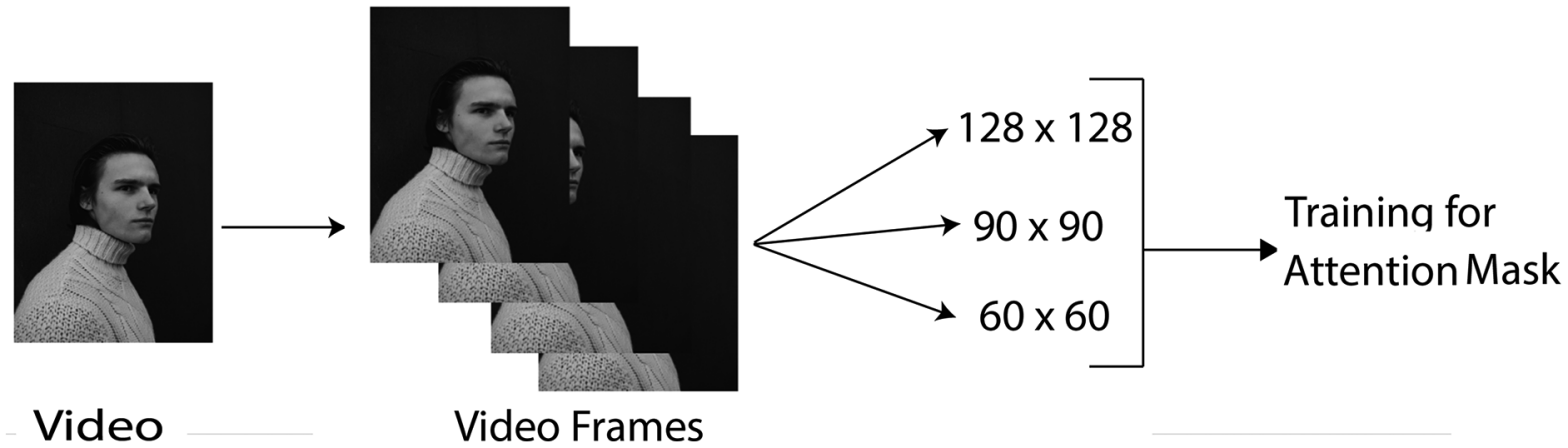
Data pre-processing. Image credit: James, https://www.pexels.com/photo/young-man-in-turtleneck-sweater-11682017/.

The data preprocessing step converts raw data into an arranged and manageable data format. Figure 1 shows the preprocessing steps carried out. The following preprocessing steps are performed on the dataset:

1. ***Converting videos to frames:*** The Data Loader utility loads the video and converts the video into a frame array. Then all the frames of video are passed as an array into the 3D CNN.
2. ***Resizing frames:*** The frames are resized from the original size to different sizes, such as 128 × 128, 60 × 60, and 90 × 90, as shown in Fig. 1. This process is performed using the PyTorch Data Loader function. The reason for this preprocessing step is that the larger the frame size, the more it takes to complete the iterations. Thus, resizing operations makes training more effective. Further, the robustness of the proposed approach could be checked by testing on different input sizes.

### Architecture

**Figure 2:**
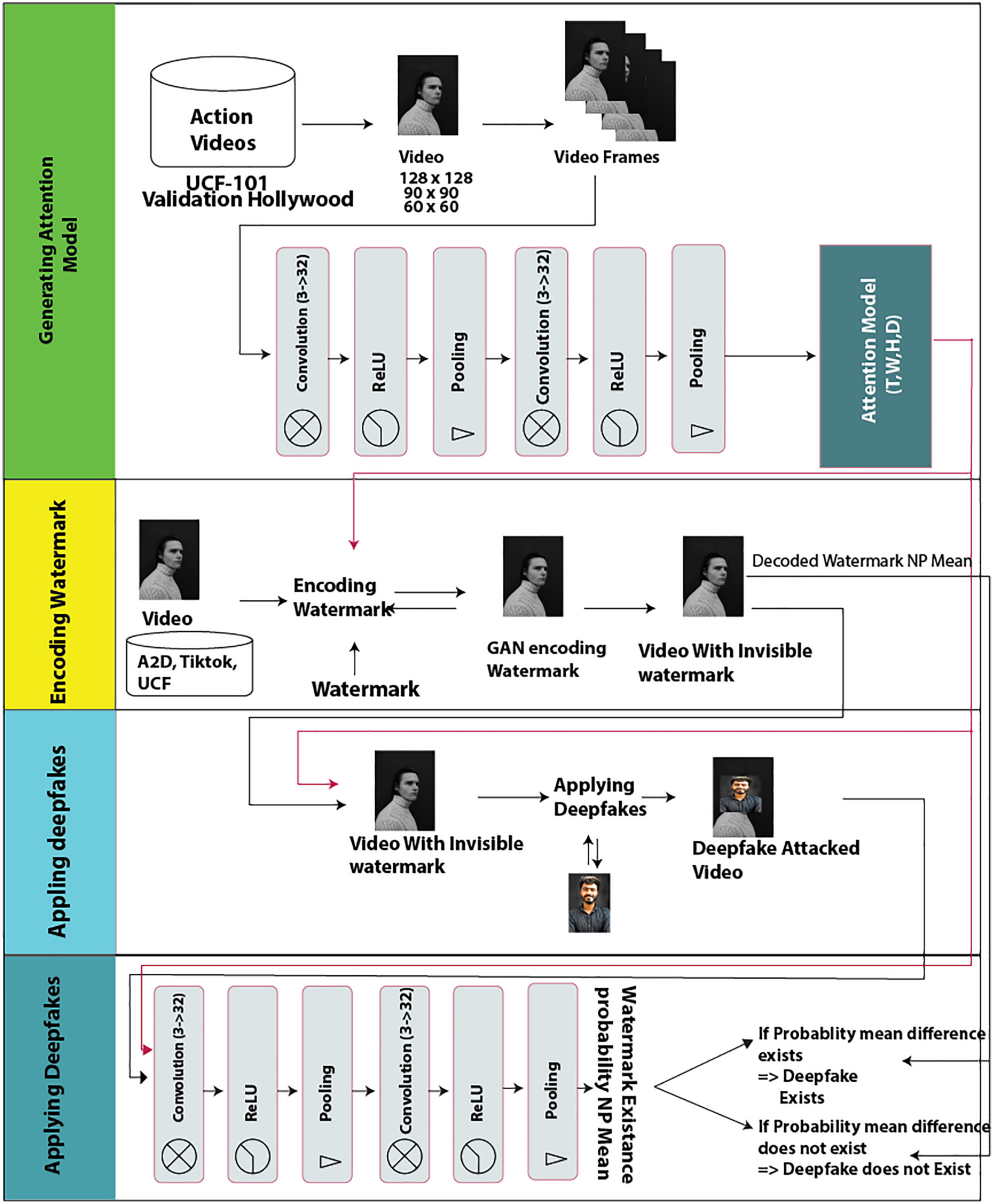
Proposed model graphical architecture. Image credit: James, https://www.pexels.com/photo/young-man-in-turtleneck-sweater-11682017/.

The model consists of a CNN and two different deep neural networks called the encoder and decoder networks. Its architecture is shown in Fig. 2. The four stages or processes of architecture are explained in the following sub-segments.

#### Attention model generation

The input video dataset is first fed to a 3D convolutional neural network, which has the same characteristics as the proposed encoder and decoder networks. This CNN is trained on the UCF101 dataset to generate an “attention model” or “attention mask”. The “attention mask” is the feature network trained to detect different scenes. It is represented as (T, W, H, D), where T = Tensor, W = Width, H = Height, and D = Data Dimension. The attention generated from this CNN is further used by the encoder network to encode watermarks into video. Both the encoder and the decoder share the same network to encode and decode watermarks.

The 3D convolutional network consists of two hidden layers separated by ReLU and batches of normalised 3D layers. Both 3D convolutional layers use 32 output channels and three input channels. The ReLU activation function is used with 3D batch normalisation of 32 layers. This method is an extension of RivaGAN (Zhang et al., 2019). Further details can be found in “Evaluation metrics” of RivaGAN (Zhang et al., 2019).

The UCF101 dataset is used because it has more actions than Hollywood. Moreover, the ReLU activation function instead of sigmoid is used for faster processing of the dataset with frame sizes copped to different dimensions, *i.e*., 160 × 160, 90 × 90, and 128 × 128. The network is trained over different epochs (11 to 125 epochs).

As an optimizer, the Adam Optimization function is used with a learning rate of 0.0005 and with a kernel size of (1, 11, 11) and padding of (0, 5, 5). Further, many mini-batch sizes are used, *i.e*., 12, 15 to 100, but we’ve got good results with mini-batch size 32. We have trained our network for 4 h to 9 h on Kaggle 16 GB GPU to get 99% accuracy. Table 3 shows details of the network parameters.

**Table 3:** Details of network parameters.

Network parameters	values
Total parameters	589,510
Trainable parameters	589,510
Nontrainable parameters	0
Learning rate	0.0005
Optimizer	Adam
Epochs	75
Iteration per epoch	417

#### Encoding watermark

In the encoding phase, the attention and watermark are passed to the generative network for training to match the attention mask of (T, W, H, D) of the video frames. A generated watermark is infused and matched to the scene of the video. Hence, the watermark is not visible.

#### Decoding watermark

To decode the watermark, the same “attention mask” is provided along with the video. Both decoder and encoder are trained with the same “attention mask”, hence the decoder network knows from the video and the attention from where to find the watermark. The decoder network decodes a watermark from the frame and returns the probability of the watermark in the frame.

#### Identification process (applying deepfakes)

As shown in Fig. 2, the decoding watermark and the “attention mask” are both needed to apply a deepfake. Hence, if an attacker does not have our attention model then he cannot decode the encoded watermark and, consequently, cannot apply deepfake on video. On supplying the “attention mask” for successful decoding of the watermark, if the probability mean of the watermark array of 32 elements remains the same, it is assumed that the video is real and not affected by a deepfake attack. We can check if the video is affected by the deepfake attack or not by checking the difference between the probabilities mean calculated before the deepfake attack and after the deepfake attack. When a deep-fake attack is successfully prevented, the before and after probability mean difference value is zero. Whereas if the video is affected by the deepfake attack, then after decoding the watermark output probability mean is different than the real video probability mean, *i.e*., the probability mean before and after the deepfake attack would be changed and their difference would be non-zero. Thus, the proposed model can be used not only to prevent deepfake attacks but can also be used to detect deepfake applications.

### Train test strategy

In phase 1, CNN is trained using UCF101 to learn action features and to generate an “attention mask.” In phase 2 and phase 3 of the proposed approach, the UCF101 (Soomro, Zamir & Shah, 2012) dataset is used to train the encoder and decoder networks for embedding watermarks into video frames such that those watermarks aren’t visible. The data consists of videos offering 101 different actions. 100% of the samples of UCF101 are used as a training sample, while we’ve used different datasets such as Hollywood, A2D, and TikTok for validation or testing purposes. 10% of the Hollywood dataset (Marszałek, Laptev & Schmid, 2009), A2D (Xu et al., 2015), and TikTok (van de Ven, 2020) video datasets are used as validation sets for the encoder and decoder. The training set is used to train and fit the model, while the testing set is used to test and evaluate the model.

After completing the training phase, testing for embedding the watermark is done. In this phase, watermarks are embedded in different datasets. Those watermarks are decoded, and after decoding the watermark, we get the probability of the watermark. Again, in phase 4, A2D and TikTok videos are used to test the deepfake attack application. Watermarked videos are then subjected to applying deepfakes using DeepFaceLab Software. Applying deepfake on a video clip takes almost 4–9 h of training time on a 4 GB GTX 1650 NVidia GPU. When decoding the watermark from those videos, the probability of the watermark becomes ~0. The training and testing results of the proposed approach are reported in the next section.

### Experimentation setup

In the experimentation setup, we have trained the encoder-decoder network on a 16 GB GPU and 14 GB of RAM on Linux-based environment with Python 3.8. To encode videos, decode watermarks, and apply deepfakes, Python 3.8 on a 4 GB NVidia 1650 GTX with a core i5 9th generation CPU and 16 GB of RAM on Windows 10 is used.

## Results

### Evaluation metrics

The following evaluation metrics are used to evaluate the performance and results of the proposed model:

**True Positive:** The real positive predictions that are identified as true are called true positives.

(4.1) $${\rm TP} = \displaystyle{{{\rm TP}} \over {\left( {{\rm FN} + {\rm TP}} \right)}}$$

**True Negative:** The negatives that are correctly predicted and identified in the evaluation are called true negatives.

(4.2) $${\rm TN} = \displaystyle{{{\rm TN}} \over {\left( {{\rm FP} + {\rm TN}} \right)}}$$

**False Positive:** The positive predictions that are wrongly identified or predicted.

(4.3) $$\displaystyle{{{\rm FP}} \over N} = \displaystyle{{{\rm FP}} \over {\left( {{\rm FP} + {\rm TN}} \right)}}$$

**False Negative:** The negative predictions that are wrongly identified or predicted.

(4.4) $${\rm FN} = \displaystyle{{{\rm FN}} \over {\left( {{\rm FN} + {\rm TP}} \right)}}$$

**Accuracy:** Accuracy is the amount of correctly identified predictions from the total number of predictions. Accuracy is a good metric in case the dataset is imbalanced.

(4.5) $$Accuracy = \; \displaystyle{{{\rm TN} + {\rm TP}} \over {\left( {{\rm TN} + {\rm FN} + {\rm FP} + {\rm TP}} \right)}}$$

**Precision:** It is the positive interpretation of accurately predicted total positive interpretations.

(4.6) $$Pr = \; \displaystyle{{{\rm TP}} \over {\left( {{\rm TP} + {\rm FP}} \right)}}$$

**SSIM:** Structural similarity index (SSIM) shows how much an image is degraded. In this case, when the watermark is embedded into the video frames, SSIM shows how many images will be distorted by the watermark.

(4.7) $${\rm SSIM}\left( {{\rm x},{\rm y}} \right) = {\rm \; }\displaystyle{{\left( {2{{\rm \mu }_{\rm x}}2{{\rm \mu }_{\rm y}} + {\rm c}1} \right)\left( {2{{\rm \sigma }_{{\rm xy}}} + {\rm c}2} \right)} \over {\left( {{\rm \mu }_{\rm x}^2 + {\rm \mu }_{\rm y}^2 + {\rm c}1} \right)\left( {{\rm \sigma }_{\rm x}^2 + {\rm \sigma }_{\rm y}^2 + {\rm c}2} \right)}}$$

where ${{\rm \mu }_{\rm x}}$ is an average of x, ${{\rm \mu }_{\rm y}}$ is an average of y, ${\rm \sigma }_{\rm x}^2$ is the variance of x, and ${\rm \sigma }_{\rm y}^2$ is the variance of y.

**PSNR:** Peak signal to noise ratio (PSNR) is the ratio between distorting noise power and the possible maximum signal value. This method provides the image quality index. This value gives the quality of the generated output image by the trained encoder and decoder network.

(4.8) $${\rm PSNR} = 20\log 10{\rm \; }\left( {\displaystyle{{{\rm MAXf}} \over {\sqrt {{\rm MSE}} }}} \right)$$

where MAXf is max signal value and MSE is the mean square error.

**Probability:** Probability gives the likelihood of happening or existence of an event. In our proposed model probability gives the chance of encoded elements of a watermark array in the video frame.

(4.9) $${\rm P} = {\rm \; }\displaystyle{{{\rm Outcome\; }} \over {{\rm Total\; Outcomes}}}$$

**Mean Square Error:** It is the average squared difference between estimated values and actual values.

(4.10) $$MSE = \; \displaystyle{1 \over {\rm n}}\mathop \sum \nolimits_{i = 1}^n {\left({{Y_i} - \hat{Y}_i} \right)^2}$$

where Y is the observed values vector, ${\hat Y}$ is predicted values and n is the total predictions.

### Ablation study

The performance of the proposed encoder-decoder network is measured on the training parameters of noise, attention parameters of the optimizer, activation function, batch sizes, and number of epochs. While the encoding and decoding of watermarks are measured by the probability returned by the network on decoding after encoding an array of digits, the encoder and decoder are first trained on the UCF101 Action Recognition dataset (Soomro, Zamir & Shah, 2012) with a variation of the validation dataset. The network is trained with a 100% training UCF101 action dataset and with the 10% Hollywood2 validation dataset. In a previous study, a CNN was trained on the Hollywood2 dataset using the sigmoid activation function for 300 epochs over 2 to 3 days (Zhang et al., 2019). The network is also evaluated using different activation functions with a variation of optimizers, epochs, and learning rates.

***Optimization:*** An optimizer is used to tune parameters and to minimise the cost function of the neural network. It is an important aspect that makes a difference in training effectiveness. There are a variety of optimizers available. Therefore, to ensure the network’s effectiveness, training results with different optimizers are compared. The encoder-decoder network is trained on the Adam optimizer and the SGD optimizer. The Adam optimizer was found to be a good fit for training encoder-decoder.

***The Learning Rate:*** This parameter defines how many threshold weights will be updated to obtain optimal weights. Our encoder and decoder network seem to extract features from the video at the rate of 5e−4.

***The Batch Size:*** A dataset cannot be passed through a neural network in one batch. So, the dataset is divided into mini samples called “batches.” Those batches are fed into the network one by one. In this study, a huge dataset of videos is used, from which frames must be extracted and the encoder-decoder network has to be trained over these. To cope with the available resources and get the best results, we had to use 32 batches per iteration.

***Epochs:*** When one entire dataset is passed backward and forward through the encoder and decoder network once, it will be considered one epoch. We have divided the dataset into several batches because the video dataset is huge and feeding all the data at once to the 3D Neural Network overflows the RAM and GPU. Only 32 videos per iteration are fed into the network. This makes a total of 417 iterations per epoch. We have run multiple epochs to compare and get the best results possible.

***Iterations:*** To complete an epoch, datasets are divided into mini-batches as discussed above. Those batches are fed into the 3D neural network one by one. Feeding one batch into NN caused one iteration to complete. In encoder-decoder training, 417 iterations per epoch are completed.

***Activation Function:*** The activation function controls the output of the layer. In this encoder-decoder network, we have tested the network on two activation functions, *i.e*., ReLU and Sigmoid. ReLU increases the learning rate and requires fewer resources than the sigmoid activation function. ReLU gives better results.

**Figure 3:**
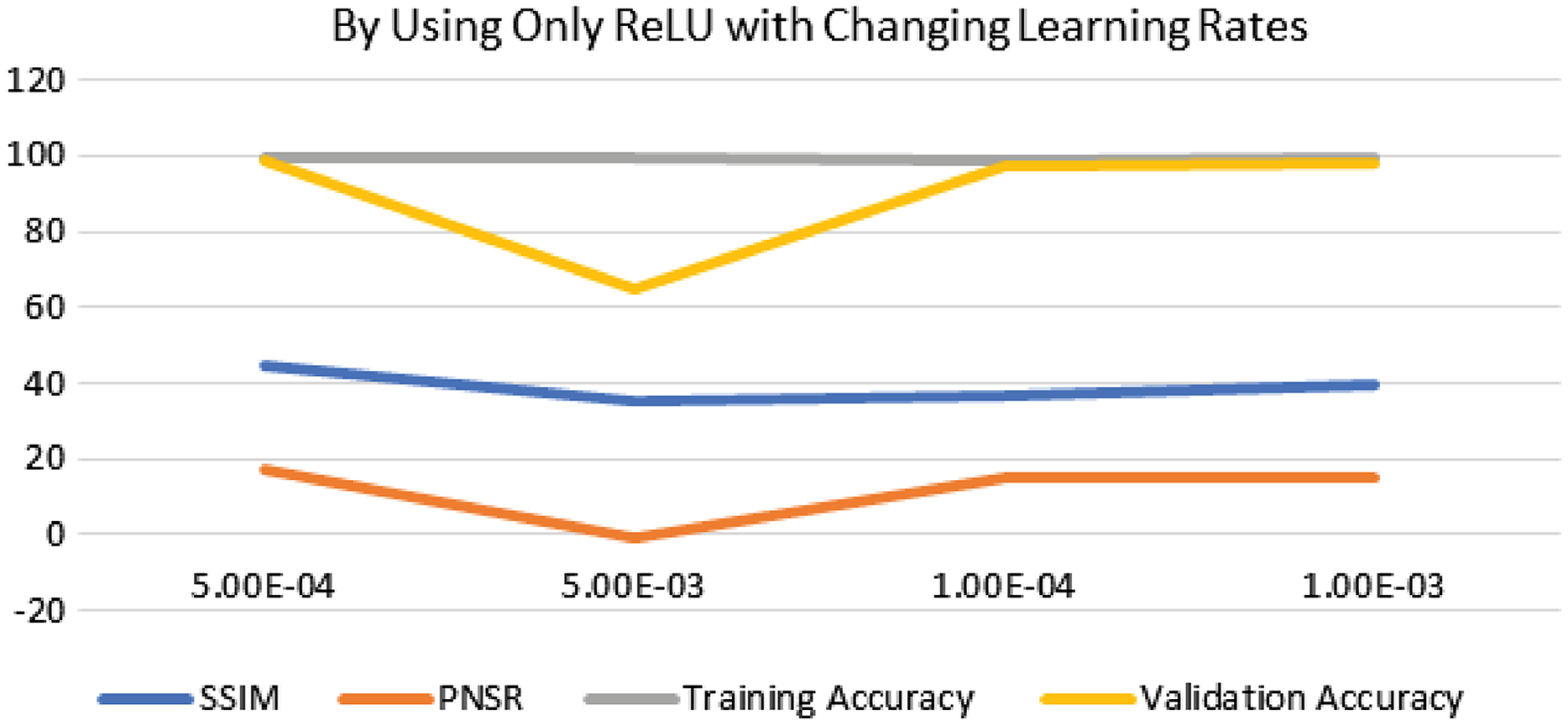
Visualization of ReLU performance concerning learning rates.

**Figure 4:**
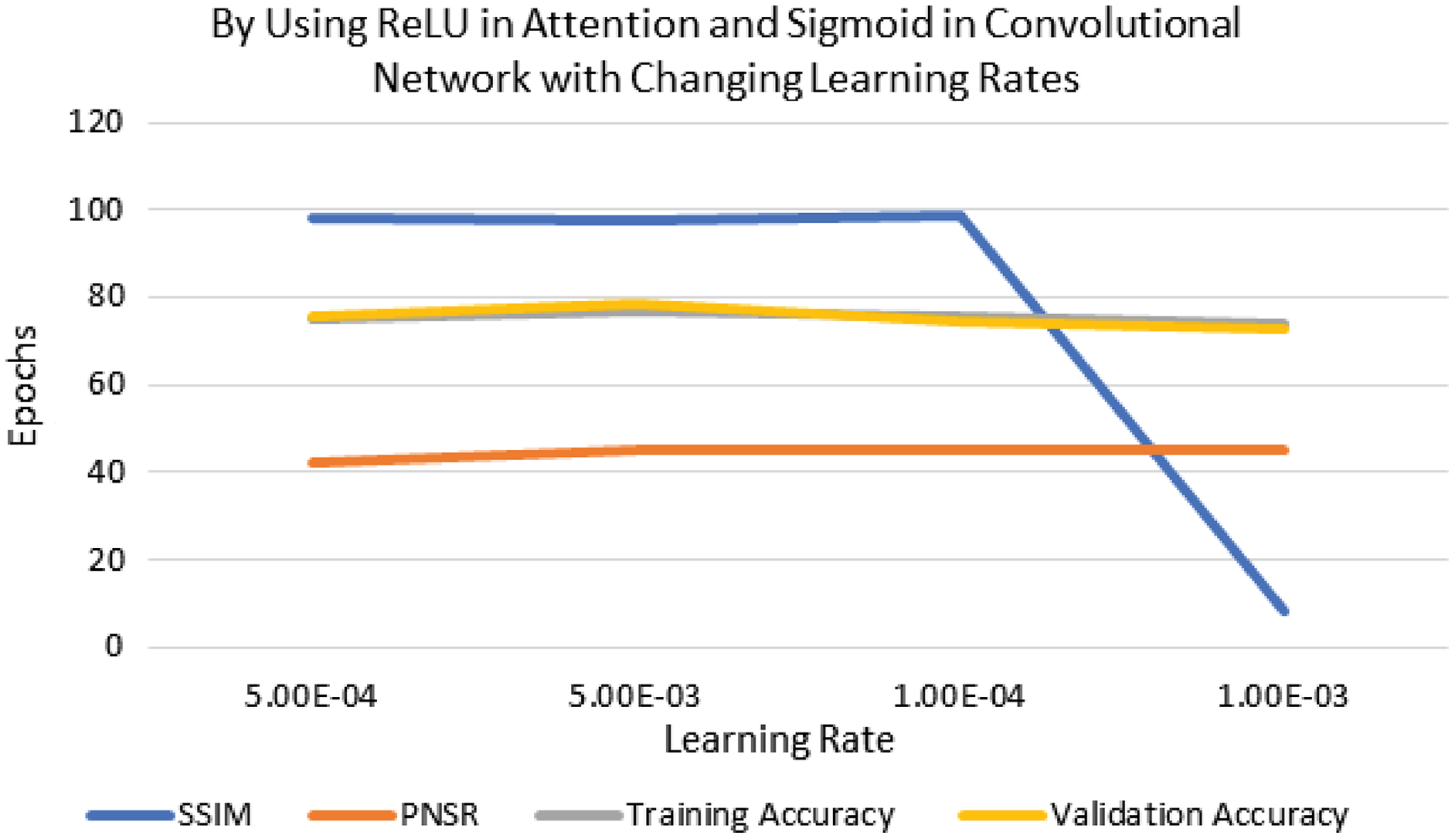
Visualization of ReLU in attention and sigmoid in CNN for different learning rates.

**Figure 5:**
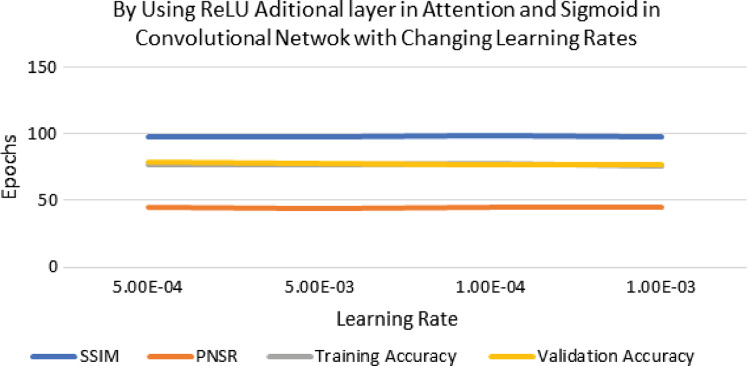
Visualization of ReLU with extra layer in attention and sigmoid in CNN using different learning rates.

**Figure 6:**
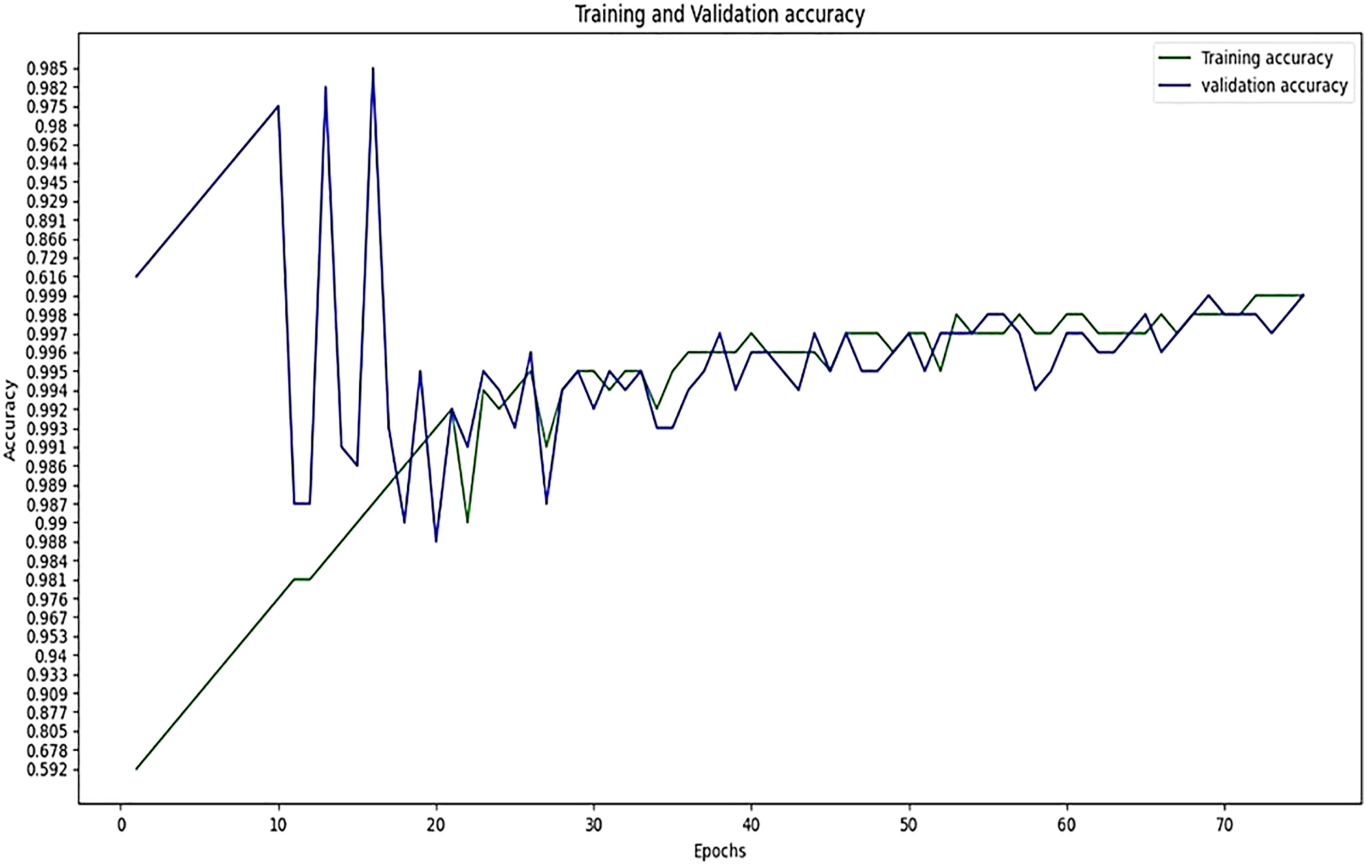
Training accuracy *vs* validation accuracy.

The performance of experimentation with Sigmoid and ReLU activation functions is compared in Table 4. Table 4 shows that ReLU provided an equal performance but with a faster learning rate in less time, hence ReLU performed the best. It shows the training of the encoder-decoder network over different variations of activation functions and epochs with attention. UCF101 training and Hollywood validation datasets are used to evaluate the accuracy of the ReLU activation function in both convolution and attention. The same has been tested at different dimensions. An increase in training time has been seen with the increase in the dimension of the frame of the video and an increase in accuracy alongside. The convolutional layers of the encoder-decoder network are trained along with the attention to encode and decode watermarks into the video frames by embedding watermarks into the features of the network. The strength of embedding is increased by adding extra layers, but training time is also increased by adding the new layer. An extra layer of the 3D convolutional network is added with the ReLU activation function in the whole attention network. Table 5 also shows the results of training the ReLU activation function on attention with sigmoid activation in the encoder-decoder network with a variety of different learning rates. The sigmoid activation function is used in the convolutional layer. It took 9 h with 32 epochs. Tables 6–8 show the results from all variations of the learning rate based on Table 5 activation and layers. Two layers of attention were also trained on Leaky ReLU Activation and the encoder-decoder layer was trained for 8 h, 36 min, with 40 epochs. The same configuration with the ReLU activation function has also been used. The best results are generated using only the ReLU activation function, which is shown in Table 8. A Motion Joint Photographic Expert Group (MJPEG) validation accuracy of 99.5% is acquired with good image quality after training for 56 epochs in 12 h. Details of parameters used in tuning are provided in Table 9. Figures 3–5 show the visual representation of the results generated from the variation of learning rates. The training and validation accuracy plot is shown in Fig. 6.

**Table 4:** Summary of ablation study results demonstrating evaluation time and performance metrics.

Execution on Kaggle 16 GB GPU and 13 GB RAM						
Model	Epochs	Execution time	SSIM	PSNR	Training accuracy (%)	Validation accuracy (%)
Extra Layer + Attention with ReLU and Sigmoid in CNN	32	9 h	0.981	45.057	78.7	81.3
Attention with Leaky Relu + Sigmoid Convolutional Network	40	8 h 36 min	0.98	45	76	78
ReLU attention + Sigmoid CNN	40	8 h 34 min	0.98	45.284	75	75.8
Only ReLU for both attention and CNN	56	12 h	0.301	13.988	99.8	99.5

**Table 5:** Sigmoid and CNN’s attention using different learning rates.

Learning rate	SSIM	PSNR	Training accuracy (%)	Validation accuracy (%)
5e−4	0.98	45.284	75	75.8
5e−3	0.978	44.948	77	78.3
1e−4	0.987	45.233	75.4	74.7
1e−3	0.083	44.95	74.1	72.5

**Table 6:** Extra layer in attention with ReLU and CNN with sigmoid.

Learning rate	SSIM	PSNR	Training accuracy (%)	Validation accuracy (%)
5e−4	0.98	45.121	77.1	78.4
5e−3	0.982	45.041	76.4	78.2
1e−4	0.985	45.218	77.5	76.9
1e−3	0.98	45.101	75.4	76.9

**Table 7:** ReLU in attention and CNN encoder decoder.

Learning rate	SSIM	PSNR	Training accuracy (%)	Validation accuracy (%)
5e−4	0.446	16.966	99.7	99.2
5e−3	0.351	−1.095	99.7	64.7
1e−4	0.371	15.058	99.2	97.2
1e−3	0.398	15.198	99.7	98

**Table 8:** Detailed evaluation of different combinations of parameters.

Sr. No	Activation function	Dataset	Dimensions	Epochs	Execution time	TL	TRA	MA	SSIM	PSNR	VCA	VSA	VMA
1	Sigmoid	UCF-101	160 × 160	11	4 h	27.9	94.5	95.9	95.6	42.092	98.1	95.9	98.4
2	Sigmoid	UCF-101 Clipped	160 × 160	50	9 h	0.441	90.5	92.5	95.9	42.05	89.3	88.2	93.5
3	Sigmoid + One Extra Hidden Layer	UCF-101	160 × 160	15	4 h	13.75	53.9	54.6	97.4	41.9	53.6	53.3	53.6
4	Sigmoid	UCF-Clipped	90 × 90	125	8 h 8 min	33.1	93.7	93.5	95.7	42.136	86.1	84.2	93.2
5	ReLU	UCF-Clipped	128 × 128	75	8 h 50 min	1.6	99.6	99.9	46.8	17.11	99.2	99.7	99.9
6	ReLU	UCF-Complete	90 × 90	25	5 h 20 min	3.5	99.1	99.6	50.1	18.102	96.6	98.9	99.7
7	ReLU	UCF-Complete	128 × 128	20	8 h 9 min	2.7	99.3	99.7	38.9	16.474	95.5	97.8	98.4
8	ReLU	UCF-Complete	90 × 90	25	5 h 22 min	3.8	99	99.6	47.23	16.677	91.7	96.9	98.6
9	ReLU	UCF-Complete	90 × 90	40	8 h 34 min	2.7	99.3	99.7	40.8	15.357	96.8	98.6	99.2
10	ReLU	UCF-Complete	90 × 90	56	12 h	0.016	99.6	99.8	0.301	13.988	97.4	99.3	99.5

**Note:**

This table represents further ablation study. We have tested our network on frame sizes, and activation functions. This table shows 56 epochs with 90 × 90 dimensions in the input frame giving faster and better results. TL, Training Loss; TRA, Training Raw Accuracy; MA, MJPEG Accuracy; SSIM, Structural Similarity index; PSNR, Point Scale Noise Reduction; VCA, Validation Crop Accuracy; VSA, Validation Scale Accuracy; VMA, Validation MJPEG Accuracy.

**Table 9:** Results using different parameter values.

Sr. No	Video sequence	Length (S)	Input type/Frames	GAN iterations	PBAD/Probablity mean before deepfake	PAAD/Probability mean after deepfake	Attack prevention
1	TikTok1-Person talking still	79	Mp4/1,581	2,229	3.4935982	3.3127096	Yes
2	TikTok2-Girl doing yoga	24	Mp4/494	963	4.4875568	4.4758565	Yes
3	TikTok3-Person praying	20	Mp4/400	104	3.2043445	3.1146355	Yes
4	Tiktok4-Girl throwing balloon	29	Mp4/580	1,502	3.3256598	3.2368836	Yes
5	Tiktok5-Girl posing	13	Mp4/260	2,285	3.7952585	3.7883663	Yes
6	Tiktok6-Person catching massage	22	Mp4/440	1,120	1.8958532	1.8822329	Yes
7	Tiktok7-Person facing mirror	22	Mp4/440	1,126	3.2187856	3.1177864	Yes
8	Tiktok8-2 boys and 1 girl walking together	18	Mp4/360	1,315	2.8854856	2.7081008	Yes
9	UCF101-1-Girl hair cut	10	Avi/200	1,155	3.183166	3.1810246	Yes
10	A2D-1-Man eating	10	Avi/200	1,005	3.5830052	3.404806	Yes
11	A2D-2-Man throwing ball	6	Avi/121	1,750	3.8360534	3.8261929	Yes
12	A2D-3-Two persons eating	5	Avi/110	2,114	3.8250728	3.8077369	Yes
13	A2D-4-Person playing with the parrot	5	Avi/110	2,411	3.6817842	3.6800249	Yes
14	A2D-5-Person eating purge	5	Avi/110	2,511	3.7410305	3.7398624	Yes
15	A2D-5-Girl eating meal	5	Mp4/110	2,872	3.7010093	3.7002363	Yes
16	A2D-6-Man eating bun	9	MP4/175	2,930	3.9093244	3.9035048	Yes
17	A2D-7-Girl picking a boy	4	Mp4/91	3,011	4.03142	4.0215335	Yes
18	A2D-8-Two boys dipping bread and eating	5	Mp4/150	3,108	3.4474072	3.4505396	Yes
19	A2D-9-Man eating burger	9	Mp4/180	3,208	4.695134	4.6312027	Yes
20	A2D-10-Man eating competition	10	Mp4/151	3,408	1.6353282	1.629467	Yes
21	A2D-11-woman eating jam	4	Mp4/91	3,575	2.5019636	2.4771504	Yes
22	A2D-12-Man eating with a stick	7	Mp4/150	3,670	3.5874434	3.5911524	Yes
23	A2D-13-Two boys eating sticks	7	Mp4/144	3,899	3.172478	3.1686606	Yes
24	UCF-2-Girl doing makeup	6	Mp4/135	44,126	4.530153	4.542652	Yes
25	UCF-3-Girl applying lipstick	10	Mp4/229	4,226	3.2072487	3.2305214	Yes
26	UCF-4-Girl riding bike	10	Mp4/204	4,426	4.7104664	4.7176847	Yes
27	UCF-5-Woman tooth brushing	10	MP4/204	4,745	4.432059	4.437254	Yes
28	UCF-6-Playing drums	15	MP4/300	5,013	5.0981035	5.099039	Yes
29	UCF-7-Boxing person	4	Mp4/84	4,909	4.5685688	4.4587589	Yes
30	UCF-8-Exercising person	13	Mp4/270	5,000	3.4585654	3.5585465	Yes
Total frames: 8,074		Total iterations: 122,561			Success rate:		100%

## Discussion

Independent testing is performed to generate validated results. The training dataset is used to fit the model with the parameters and the weights, while the testing dataset is only used for validating the effectiveness and performance of the model after every epoch. In phase 1, 100% of the UCF101 dataset is used to train CNN to generate the “attention model.” Then 10% of the unbiased Hollywood2 dataset is used for evaluation and testing of the model. In addition, after training and validating the attention network for feature extraction, in phases 2 and 3 of the proposed approach, an encoder-decoder network is trained to embed invisible watermarks into video frames. For this purpose, UCF101 (Soomro, Zamir & Shah, 2012) is used as a training set, whereas the A2D action recognition dataset (Xu et al., 2015), TikTok trending videos (van de Ven, 2020), and the Hollywood2 dataset (Souza et al., 2011) are used as testing sets for encoder-decoder.

The watermarked videos are then decoded to check the performance of the decoder network. The outcome is the probability “P” containing 32 probabilities each for one watermark insertion. Thus, the probabilities arrays of size 32 are filled with probabilities values. Further, the mean value of probabilities is calculated, which remains the same for a single video in the case if it is not fabricated. The watermark is encoded first into the testing dataset mentioned in Table 1 and then it is decoded to get the probability mean of the watermark array of 32 elements, which remains the same when a video is real and not fabricated, after encoding watermarks.

**Figure 7:**
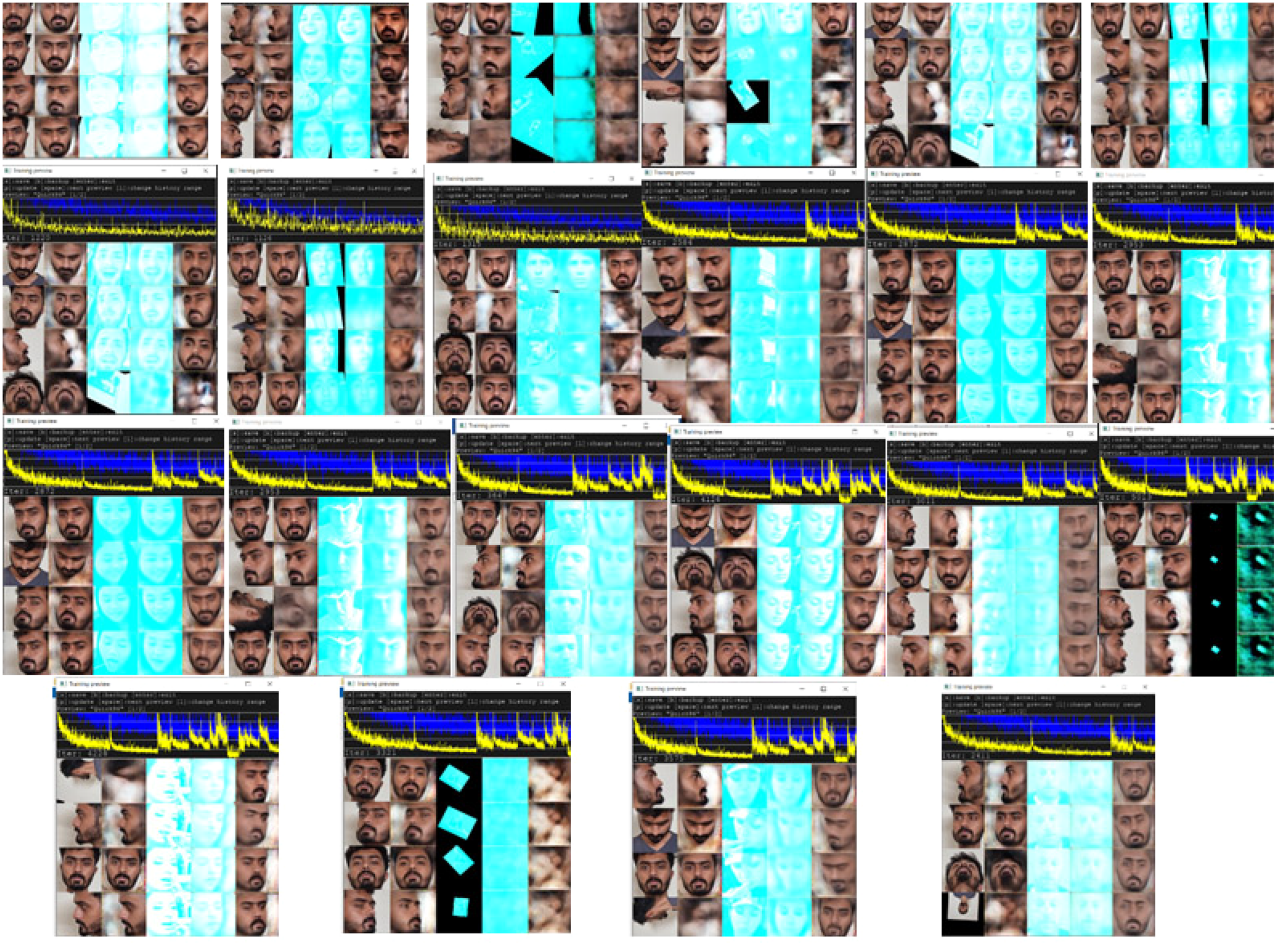
Deepfake swap training with GANs network of different videos.

Finally, in phase 4, to evaluate the effectiveness of the proposed approach to preventing deepfake attacks, the face dataset is swapped onto video datasets. Watermarked videos are subjected to applying deepfakes using DeepFaceLab Software 2.0 (iperov, 2018). Encoded watermark videos are trained with the swapping face on the GAN using DeepFaceLab 2.0 (iperov, 2018) to create fake swapped videos. A total of 30 videos from A2D, Hollywood2, and TikTok are used to test the prevention of the deepfake attack. As mentioned in Table 9, watermarks are encoded into the videos and then decoded to test the existence of deepfakes. We calculated the probabilities means of output probabilities of input elements in the video, from the decoder and compared those values with the real watermarked video’s probabilities means from phase 1. Because FaceSwap changes the probabilities of the encoded watermark throughout the frames and replaces the watermark with swapped faces, the probability mean gets disturbed and a different probability mean value is obtained for fake video when decoded. The difference suggests that deepfake was applied. Table 9 provides the details of the videos, which are first embedded with watermarks and then swapped for our video frames. The deepfake swap training process with GANs to authenticate deepfakes has been shown in Fig. 7.

***Comparison with the state of the art:*** In the field of deepfake detection and prevention, there has been a lot of research going on using traditional machine learning methods and advanced deep learning methods. A state-of-the-art comparison of different methodologies with deepfake detection, prevention, and proposed methodology is presented. Watermarks are embedded in a few prevention methods. However, these watermarks are visible. The proposed method uses an encoder-decoder network, which is trained on a 3D neural network, to embed a watermark into pixels of video frames. In this way, the watermark is hidden and cannot be decoded without an attention-trained attention mask with specific noise settings. This is a steganography technique that makes deepfake prevention more effective.

Guera & Delp (2019) used a recurrent neural network to detect deepfakes, this gives 96.7% of accuracy in detecting deepfakes. In the same way, Dordevic, Milivojevic & Gavrovska (2019) has used SIFT features, *i.e*., brightness changes, scaling, *etc*., to detect deepfakes. This gives 97.91% accuracy in detecting deepfakes DeepFaceLab. However, when creating deepfakes with DeepFaceLab 2.0 (iperov, 2018), there are many options to improve the experience of creating deepfakes with minimal loopholes, either manually or automatically. In the near future, deepfakes may surpass the accuracy of detection. There are a few methods available, like Sethi et al. (2020), which embed watermarks into the least significant bit of any video. This old watermarking technique gives approximately 100% accuracy in preventing deepfakes, while there are more methods available, such as Lv (2021), used a smart watermarking technique to prevent deepfakes. Lv (2021) suggest that their method has a 70.43% accuracy in preventing deepfakes. In their method, they have embedded a smart watermark and blur effect on a face in different positions. The watermark is applied on the least significant bit, and the smart watermark can be copied and applied again to video affected by deepfake. Lv (2021) created a smart watermarking method to embed a watermark using a convolutional structure to extract features of a face, while a deconvolutional structure generates a watermark according to the convolutional structure. The size of the watermark is minimised to the extent it is unpredictable by the human eye, while the blur of the image is manipulated to make the watermark invisible.

Alattar, Sharma & Scriven (2020) also encoded watermarks using deep learning methods by embedding a watermark into face marks. Those watermarks can be copied easily in Alattar, Sharma & Scriven (2020) as the watermark is either visible or blurred. While the smart watermarking technique (Lv, 2021) implies images and puts the watermark inside the image without encrypting it. However, in the proposed approach, watermarks are embedded into the features of the video frames. These features aren’t crackable without the presence of noise and attention trained using selective optimized parameters. This is an advanced steganography technique to embed and encode watermark in such a way that the watermark is infused into the video frames and can only be extracted with the proper parameters of training. Hence, a copy attack is not possible in this proposed methodology. A summary of results, comparisons, and limitations is given in Table 10. Table 11 shows the comparison between RivaGAN (Zhang et al., 2019) and the proposed approach. It shows that RivaGAN has high training time and it has no experimentation or implementation on deepfake prevention. It shows that RivaGAN has not experimented with their modal on deepfakes. Moreover, with less training time, the proposed model gives improved SSIM and PSNR values. It means less noise and improved output video. In Table 11, we have also reported MSE loss, which is 0.15 for training and 0.16 for validation.

**Table 10:** Summary of state-of-art comparison.

Sr. No	Year	Model	Accuracy (%)	Technique	Dataset used	Training iterations	Limitation
1	2020	Alattar, Sharma & Scriven (2020)	100	CNN, MTCNN	Custom one video	3,800	DCT watermarking method is used to prevent deepfakes. Copy attack is possible and used hashed complex mt CNN to tackle this problem.
2	2021	Lv (2021)	70.43	CNN, De Convolutional Neural network Attention	CalebA Images	-(not given)	It is used to embed mere simple watermarks in facial features only. It is not supported for video data.
3	2022	Proposed model	100	LSTM, 3D CNN, GAN,	8 UCF101 (Soomro, Zamir & Shah, 2012) videos, 13 A2D (Xu et al., 2015), 8 TikTok trending videos (van de Ven, 2020)	122,561	We have used watermark feature embedding to prevent copy attacks. Watermark is embedded into video frames and invisible to the human eye.

**Table 11:** RivaGAN and proposed methodology comparison.

Sr. No	Model	Dimension	MES loss	Training duration	PSNR	SSIM	MJPEG validation accuracy	Croped validation accuracy	Scaled validation accuracy	Videos tested on deepfakes
1	RivaGAN Attension + Noise	32	N/A	3 Days	42.61	0.960	0.997	0.995	0.987	Nill
2	Proposed Approach Attension + Noise	32	0.16	12 h	13.988	0.301	0.995	0.974	0.993	30 videos tested on Deepfakes

**Note:**

Previous and Proposed Methodology Comparison.

## Conclusions

Deepfakes have a lot of threats to society along with good usage in the entertainment industry. Due to advancements in generative models, the detection of deepfakes is not the solution, but prevention methods are the need of the domain. A deepfake attack prevention approach is presented. Prevention methods add a layer of security to video frames. Therefore, they are effective in the long run. Hidden watermarks are embedded in the features of the video frame, similar to steganography techniques. Furthermore, attention masks rely on noise and optimization of specific parameters. Hackers need this attention mask or attention mask generating model in order to decipher watermarks and apply deepfakes. Therefore, videos stored with the proposed approach are protected from possible deepfake attacks. The proposed approach has attained 99.5% of training accuracy, which is 0.1% lower than the previously trained network. However, SSIM and PSNR are 13.98 and 0.301, which are better than the previous network. We have evaluated the effectiveness of our technique by first embedding watermarks into videos from the action and trending social media video dataset. The proposed approach was reported to be 100% effective in the prevention of deepfakes. The future work plan for the research is to improve the watermarking technique using LSTM to train encoder-decoder networks with different available action recognition datasets to make a model to embed watermarks on all available scenes and to continue the work on audio deepfake prevention.

## Supplemental Information

10.7717/peerj-cs.1125/supp-1 Supplemental Information 1 Code of approach and dataset links.

## Additional Information and Declarations

### Data Availability

The following information was supplied regarding data availability: (1) The python source code project, all raw code, data .py files, and configuration files are available with code at GitHub: https://github.com/shahidmuneer/deepfakes-watermarking-technique. (2) The UCF101 dataset is available at UCF: https://www.crcv.ucf.edu/data/UCF101.php. (3) A2D dataset is available at the University of Michigan: https://web.eecs.umich.edu/~jjcorso/r/a2d/index.html#downloads. (4) Hollywood2 dataset is available at IRISA/INRIA Rennes France: https://www.di.ens.fr/~laptev/actions/hollywood2/. (5) TikTok dataset is available at Kaggle: https://www.kaggle.com/datasets/yasaminjafarian/tiktokdataset.
