# Supplementary material for: Deepfake attack prevention using steganography GANs
Source: PeerJ Comput Sci. 2022 Oct 20;8:e1125. doi: 10.7717/peerj-cs.1125 (PMC9680891; doi:10.7717/peerj-cs.1125)
Supplement: Supplemental Information 1 [file peerj-cs-08-1125-s001.zip › deepfakes-watermarking-technique-master/datasets_links.html]

version https://git-lfs.github.com/spec/v1
oid sha256:94d6a3fef2f9c159959bd40fccaa5e205fff209ffea0e7388b3261bd1444b7f5
size 337
